# The Mediating Role of the Principal–Teacher Relationship in Innovative School Leadership and Teacher Professional Learning According to Turkish Teachers’ Perceptions

**DOI:** 10.3390/bs15040450

**Published:** 2025-04-01

**Authors:** Mehmet Özdoğru, Yurdagül Doğuş, Muhammet İbrahim Akyürek

**Affiliations:** 1 Department of Special Education, Kütahya Dumlupınar University, Kütahya 43100, Türkiye; 2 Department of Educational Administration, Kocaeli University, İzmit 41001, Türkiye; yurdagul.dogus@kocaeli.edu.tr; 3 Department of Educational Administration, Selçuk University, Konya 42250, Türkiye; m.ibrahimakyurek@gmail.com

**Keywords:** innovative school leadership, teacher professional learning, principal–teacher relationship, Türkiye

## Abstract

The relationship between teacher professional learning and school leadership has recently attracted great attention from scientists. However, only a few studies have focused on the relationship between innovative school leadership and teacher professional learning. Therefore, this study aimed to draw attention to the mediating role of the principal–teacher relationship in the relationship between innovative school leadership and teacher professional learning. The study used a quantitative method with a correlational, cross-sectional, and mediation design. Data obtained from 357 teachers working in high schools in Eskişehir, Türkiye, were examined with a mediation analysis based on the bootstrap method. The findings showed that teachers perceived their professional learning highly, school administrators perceived innovative school leadership characteristics highly, and principal–teacher relationships had a significant effect. In addition, moderate positive significant relationships were determined between innovative school leadership and teacher professional learning and principal–teacher relationships; low positive significant relationships were determined between teacher professional learning and principal–teacher relationships. It was determined that the principal–teacher relationship mediated innovative school leadership and teacher professional learning. Our results expand the field’s understanding that innovative leadership is practiced in Türkiye, an eastern society, and that school leadership has the potential to improve student outcomes indirectly through teacher professional learning.

## 1. Introduction

Technological developments such as artificial intelligence that have left their mark on the era and the pressure to raise students as individuals with 21st century skills necessitate that schools that educate the new generation be managed with an innovative leadership approach that is compatible with these developments and foresees the present and the future (Davis, 2019; Fios et al., 2024; Marron & Cunniff, 2014; Volti & Croissant, 2024). The process of change that is being experienced directs both developed and developing countries to reform their education systems and improve student outcomes in order to train their citizens as qualified human resources (MoNE, 2017; OECD, 2007). The strong conclusions of educational administration and leadership research that teachers and school principals are two indispensable factors in improving student outcomes (L. Li et al., 2016; Liu et al., 2016; S. Özdemir et al., 2023b; Thien & Yeap, 2023) make it more evident than ever that schools need school leaders who can establish positive relationships with teachers, as well as teachers who have improved their qualifications through professional learning, and thus improve student outcomes (Akyürek & Karabay, 2022; Khanthap, 2022; Kwakman, 2003; Liu & Hallinger, 2024; Özdoğru & Sarıer, 2024; Valenzuela & Callo, 2024).

It is known that teacher education is an important way to improve student outcomes (L. Li et al., 2016; Orphanos & Orr, 2014; Thien & Yeap, 2023). Therefore, it is widely accepted that teachers should be lifelong learners in order to improve student outcomes and maintain quality in education (Kwakman, 2003; Liu et al., 2016; MoNE, 2017; OECD, 2019). However, in many countries, teacher education is still mostly carried out through activities that are not aligned with the needs of teachers and contribute little to nothing to student outcomes (Liu et al., 2016; Pan & Chen, 2021; Thien & Yeap, 2023). However, studies examining innovative approaches that direct teacher education towards teacher professional learning (TPL) and provide practical skills provide striking results about the strategic importance of these approaches to TPL (Hilal et al., 2022; L. Li et al., 2016; Hallinger & Kulophas, 2019; Kwakman, 2003; Thien & Yeap, 2023). This strategic importance has led to a policy change regarding teacher learning, especially in countries with poor performance in international student comparisons (e.g., Programme for International Student Assessment [PISA]) such as Türkiye (Hilal et al., 2022; MoNE, 2017; Thien et al., 2023). It has also directed the interest of researchers to examine how leadership interacts with teacher learning (Orphanos & Orr, 2014).

The literature is rich in research showing the direct and indirect relationships of TPL with leadership (Liu & Hallinger, 2024; Liu & Yin, 2023; Liu et al., 2016; Pan & Chen, 2021; Shengnan & Hallinger, 2021; Talebizadeh et al., 2021; Thien & Yeap, 2023; Thien et al., 2024; Thoonen et al., 2011). Academicians working in the field of educational administration (Bellibaş & Gümüş, 2023; Bush, 2024; Karacabey et al., 2020; Liu et al., 2016; Talebizadeh et al., 2021; Thien & Yeap, 2023) also point out that existing literature proves the link between teacher qualifications and student learning and that school leadership plays a critical role in this link. In addition, research shows that teacher trust (Karacabey et al., 2020; L. Li et al., 2016; Liu et al., 2016; Talebizadeh et al., 2021), agency (Liu et al., 2016), responsibility (Liu & Hallinger, 2024), participation in the decision-making process (Liu & Yin, 2023), sharing information (Talebizadeh et al., 2021), and communication (Thien et al., 2024) are effective as mediators and moderators in the relationship between leadership and TPL. It can be said that all these effects point to relational processes that can be characterized by an emotional bond, in other words, principal–teacher relationships (PTR).

PTR, which is mostly explained by using the Leader–Member Exchange (LMX) theory (Zee et al., 2023), characterizes the positive dialogue and cooperation between school principals and teachers based on trust, love, and respect (Akyürek & Akkoyun, 2023). This positive dialogue and cooperation based on trust, which is an important factor in ensuring the effectiveness of schools and improving student outcomes, serves as a potential explanatory mechanism linking leadership and teacher learning (L. Li et al., 2016; Liu et al., 2016). Although the ultimate goal is to improve student outcomes (N. Özdemir et al., 2023a), PTR produces multifaceted results due to the nature of the relationships. In addition, school principals gain unique competencies such as strengthening their leadership effects and being able to enter the teachers’ acceptance zone more (Liu & Yin, 2023; Shengnan & Hallinger, 2021; Thien & Yeap, 2023).

School leaders differ from the leaders of most organizations because they lead their colleagues and an organization whose population is mostly composed of non-adult individuals (students) (Zee et al., 2023). Therefore, it can be said that within such an organizational structure, innovative leaders going beyond the norms (Davis, 2019), using transformative, ethical, and accountable practices (Ariratana et al., 2019; Valenzuela & Callo, 2024), have the potential to contribute to the ultimate goals of schools. Although theoretical (Alharbi, 2021; Davis, 2019) and empirical studies on innovative school leadership (ISL) are quite limited, the available information shows that ISL has a positive relationship with the success culture of employees (Al-Omari, 2024), the entrepreneurial behavior of teachers (Akyürek & Göktaş, 2023), cooperation and satisfaction levels (Orphanos & Orr, 2014), job satisfaction (Ghodang, 2021), performance (Ghodang, 2021; Valenzuela & Callo, 2024), and professional learning activities (Khanthap, 2022; Valenzuela & Callo, 2024).

Despite the rich knowledge explaining the relationship between school leadership and TPL, there are still some unclear areas (Liu & Hallinger, 2024; N. Özdemir et al., 2023a). Although researchers increasingly recognize the positive outcomes of school leaders’ ethical (Liu & Yin, 2023), transformational (Karacabey et al., 2020), and especially learning-centered leadership (Karacabey et al., 2020; Liu & Hallinger, 2024; Liu et al., 2016; N. Özdemir et al., 2023a; Shengnan & Hallinger, 2021; Talebizadeh et al., 2021; Thien & Yeap, 2023; Thien et al., 2024), very few studies have paid attention to the relationship between ISL and TPL (Khanthap, 2022; Valenzuela & Callo, 2024). The mediating role of PTR in this relationship is left incomplete. Therefore, we respond to the calls for research on the role of innovative leadership in individual and organizational innovations (Alharbi, 2021), exploring additional boundary conditions in the relationship between school leadership and TPL (Kwakman, 2003; Liu & Yin, 2023) and providing more information on these boundary conditions from non-Western contexts (Hallinger & Kulophas, 2019). We focus on how PTR mediates the relationship between ISL and TPL. In addition, responding to the arguments that Western societies with individualistic cultures (e.g., America, England, and Sweden) are more advantageous in terms of innovation potential (Hofstede, 1980) and that employees in these societies have more opportunities to try new practices (Kaasa & Vadi, 2010), by investigating ISL from a country like Türkiye, which is culturally characterized by high power distance and low openness to uncertainty (Hofstede, 1980), we can make a unique contribution to the existing literature.

### 1.1. Theoretical Framework and Hypothesis

**Figure 1:**
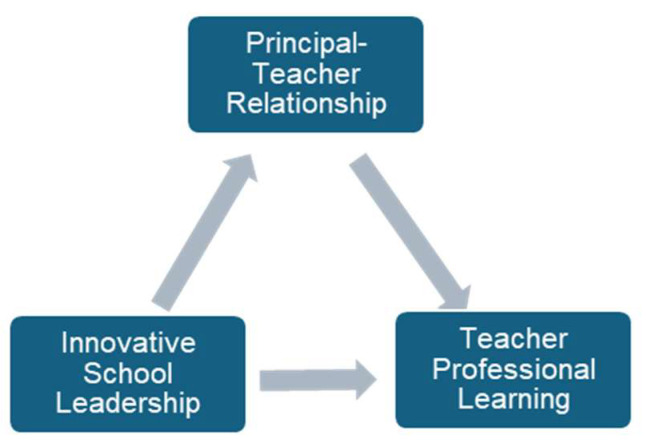
Conceptual model.

The theoretical framework of this study is built on LMX, Attachment and Path-Goal theories. According to the Path-Goal Theory, employees are satisfied with their leaders when they believe that they will be successful and when they believe that their leaders will satisfy them (House, 1971). The LMX theory also focuses on the quality of relationships and states that if the leader exhibits behaviors that will satisfy employees such as providing development opportunities, mentoring, and supporting followers, followers will feel more loyalty to their leaders and exhibit more voluntary behaviors. (Graen & Uhl-Bien, 1995). According to the Attachment Theory, which explains that this relationship is two-way, followers can establish a positive bond with their leaders as well as a negative bond. In other words, the relationship between leaders and followers can be close or conflictual (Bowlby, 1944). In this context, school principals, who play the most important role in achieving the school’s goals and ensuring its effectiveness, need to establish close relationships with teachers who are their followers while fulfilling their duties (Hoque & Raya, 2023; Zee et al., 2023). School principals should support teachers, create development opportunities for them, and provide mentoring (Galdames-Calderón, 2023; Hilal et al., 2022). For ISLs who have adopted innovation-based school management, supporting teachers and building close relationships are particularly important (Cunniff et al., 2013; Marron & Cunniff, 2014). This is because innovations inherently carry the potential to encounter resistance (Burnes, 2004; Kondakçı et al., 2015; Wang et al., 2020). Teachers who have close relationships with their principals and are supported by their principals trust them and implement the innovations planned by their principals in the classroom (Price, 2012; Price & Moolenaar, 2015). Teachers’ positive relationships with and trust in their principals can make them more willing to participate in professional learning activities (Kwakman, 2003; Y. Li et al., 2023; Louws et al., 2017). Research also confirms this. For example, in a study conducted in Türkiye, Çoban et al. (2023) found that teachers’ trust in their principals enabled them to focus more on teaching and collaborate more with their colleagues. Alazmi and Hammad (2023) modeled the relationship between principal leadership and teacher professional learning in Kuwait and concluded that when principals increase teachers’ self-confidence and create a trusting school environment, teachers will participate more actively in professional learning activities. In this theoretical framework, our study suggests that principal–teacher relationship mediates the relationship between innovative school leadership and teacher professional learning (see Figure 1).

#### 1.1.1. Innovative School Leadership (ISL)

Global economic realities force school leaders who deal with complex problems to be more creative and innovative (Marron & Cunniff, 2014). Starting with the assumption that innovation leads organizations to success (Kahn, 2018) and that an innovative school culture is possible with a suitable leadership, the concepts of innovation and leadership are integrated in the innovative leadership approach (Alharbi, 2021; Valenzuela & Callo, 2024). Innovative leadership provides theoretical support from the path-goal theory (House, 1971), which focuses on how the leader motivates his followers to achieve set goals, and the characteristics of transformational (Bass & Riggio, 2006) and visionary leaders (Westley & Mintzberg, 1989) who inspire, empower, encourage innovation, and turn their followers into reality to achieve the goals of the organization. The leadership model of the innovative leadership approach that comes to life in schools is named as ISL in the literature (Akyürek & Karabay, 2022; Marron & Cunniff, 2014).

There are several different definitions of ISL in the literature. Marron and Cunniff (2014) characterized ISL with the concepts of heart, empathy, learning, passion, perseverance, strategy, and speed, which emphasize the personal characteristics of the leader, and stated that it distinguishes itself from other leadership models with these characteristics. Fios et al. (2024) described ISL as a multifaceted effort that requires strategic management of challenges and intentional maximization of benefits. In this study, ISL was adopted in a one-dimensional conceptual approach that Akyürek and Karabay (2022) addressed from a holistic perspective from the teacher’s perspective, emphasizing the managerial practices of school principals in the innovation center and their efforts regarding their own personal development. Innovative school leader is used in the sense of “the person who motivates and mobilizes all stakeholders of the school towards innovation” (Akyürek & Karabay, 2022).

From a historical perspective, leadership approaches can be divided into two main streams: traditional and modern approaches (Jiang, 2014). ISL is positioned in the second of these currents and is included in modern approaches (Akyürek & Karabay, 2022). Distributive leadership, instructional leadership, and transformational leadership models are among the leadership approaches that the educational literature has been interested in in recent years (Gümüş et al., 2018b). Instructional leaders strive to define the school mission, manage the curriculum, and foster a positive learning environment in the school. In line with this effort, instructional leaders prioritize framing school goals, ensuring the implementation of an effective school curriculum and monitoring student progress (Hallinger & Murphy, 1985). An innovative leader’s approach that prioritizes motivating and mobilizing all stakeholders of the school in line with innovation differs from the instructional leadership approach in this context (Akyürek & Karabay, 2022).

In contrast to instructional leadership, distributive leadership and transformational leadership emphasize innovation in the organization. Distributive leadership, which reflects an understanding in which leadership roles are distributed to the stakeholders of the school, is based on the idea that schools cannot be managed by a single leader due to the complex roles and increasing responsibilities of principals (Bolden, 2011). In this leadership model, teachers actively participate in the decision-making and management processes in schools and assume some of the roles of principals (Spillane, 2005). Transformational leadership, which originates from the theory of social change, aims for a radical transformation in organizations by changing the culture, values, and motivation of employees. In this approach, the leader becomes a role model, inspires, provides intellectual stimulation and individual support to his/her followers. Thus, they support the development of employees and try to maximize the performance of the organization (Bass, 1985; Bass & Riggio, 2006). Another leadership model that has innovation in its nature is transactional leadership. The transactional leadership approach is an exchange relationship in which the leader clearly states exactly what he or she expects from the followers (Pieterse et al., 2010). The transactional leadership approach is embodied in two behavioral dimensions. The first one is goal setting and rewards. The second is adopting active management and continuously monitoring the performance of employees and taking immediate action in case of deviations (Bass & Riggio, 2006).

Although distributive, transformational, and transactional leadership models emphasize change and transformation, this is not the same as the meaning that ISL attributes to innovation and the ways in which they orient towards innovation in their organizations. Nevertheless, an ISL’s innovation-centered approach that leverages existing ideas and transforms them into unique solutions (Davis, 2019) has the capacity to extend the idealized influence, inspirational motivational practices, intellectual stimulation, and individualized attention (Bass, 1985) of the transformational leaders with whom it shares a common origin. Some studies have also shown that transformational leaders positively affect employees’ innovative behaviors (Aydın & Erkılıç, 2020; Pieterse et al., 2010). Distributive leaders can distribute some of their roles to teachers and other stakeholders, including their roles in change and transformation processes in schools (Spillane, 2005). Similarly, an ISL supports and encourages teachers in order to increase the innovation capacity of the school and provides teachers with the opportunities they need to carry out innovative practices. However, unlike distributive leadership, an ISL is the main driver of innovation in schools and holds the leadership roles related to innovation (Akyürek & Karabay, 2022). Finally, ISL differs significantly from transactional leaders’ conceptions of control (Bass & Riggio, 2006; Pieterse et al., 2010), where innovation is achieved through some form of reward-punishment and exchange. For example, ISL sees different ideas and practices of employees as an opportunity to increase the innovation capacity of the school. They have high risk tolerance even if they involve practices that deviate from the goal, and their emotional characteristics that emphasize relationships differentiate them from transactional leadership and other types of leadership (Akyürek & Karabay, 2022; Alharbi, 2021; Ariratana et al., 2019; Marron & Cunniff, 2014; Valenzuela & Callo, 2024).

#### 1.1.2. Teacher Professional Learning (TPL)

TPL is an approach to teacher learning that involves activities such as professional learning communities, workplace learning, and peer coaching embedded in the daily life of the school, where teachers collaborate with their colleagues and receive mentorship from them (Hallinger & Kulophas, 2019; L. Li et al., 2016). Characterizing a contemporary approach to teacher learning, TPL has the advantage of providing teachers with practical skills since it is implemented in schools through teachers’ individual activities as well as their collective and social interactions (Kwakman, 2003; Liu et al., 2016; Hilal et al., 2022; Thien & Yeap, 2023). The voluminous literature in recent years has illuminated many personal and organizational factors that affect TPL (Karacabey et al., 2020; L. Li et al., 2016; Liu & Hallinger, 2024; Orphanos & Orr, 2014) and has shown the important role of TPL in improving student outcomes by increasing teacher performance (Thien et al., 2023). Due to the dynamic structure of TPL, which both affects and is affected by individual and organizational factors, Liu et al. (2016)’s multidimensional conceptualization was adopted in the study. In the study, TPL was defined as “as a form of workplace learning characterized by dynamic, ongoing, interactive exchange among teachers” (Liu et al., 2016).

#### 1.1.3. Principal–Teacher Relationship (PTR)

One of the complex tasks of school principals (Fios et al., 2024; Gonzales et al., 2024; L. Li et al., 2016) is to ensure coordination between teaching and management. PTR, which indicates an emotional bond that contributes to the smooth running of school activities, plays a key role in the coordination between management and teaching (Akyürek & Akkoyun, 2023). Indeed, the literature has a rich knowledge base showing that PTR contributes to the development of teachers and their positive organizational behaviors, and thus to the improvement of student outcomes (Akyürek & Akkoyun, 2023; Bellibaş et al., 2022; Rockstuhl et al., 2012; Vermeulen et al., 2022).

Theoretically, PTR is widely based on LMX theory (Graen & Uhl-Bien, 1995; Zee et al., 2023). LMX theory is based on the idea that leaders do not communicate with all their subordinates in the same way and do not apply the same leadership model (Dansereau et al., 1975). Researchers based on LMX theory focus on how leader–member interactions and working relationships are established and developed (Graen & Uhl-Bien, 1995). In the process, in studies conducted with LMX theory, it came to be understood that a one-dimensional structure was insufficient to explain leader–member interaction (Graen & Uhl-Bien, 1995), and the theory evolved into multidimensional structures that would explain this relationship. Therefore, in this study, PTR was conceptualized as “affective bonds between teacher and principal that are unique and reflect both positive and negative qualities”, which was considered as two-dimensional by Zee et al. (2023).

Attachment theory explains that there are positive as well as negative aspects in dyadic relationships (Bowlby, 1944). Zee et al., (2023) explained this relationship in two dimensions called “closeness” and “conflict” based on the fact that there are negative as well as positive aspects in principal–teacher relationships. The closeness dimension reflects warmth, love, and emotional security in the relationship between the principal and the teacher. Teachers who experience high levels of love, warmth, and emotional trust in the principal–teacher relationship see their principals as a source of support and trust them. This makes teachers feel more confident and effective. The conflict dimension explains negativity, incompatibility, and unpredictability in the principal–teacher relationship. Teachers who perceive their relationship with their principals as conflictual are in a state of struggle with their principals. This makes them feel exhausted and ineffective (Zee et al., 2023).

#### 1.1.4. Turkish Context

In 2023, nearly 20 million students, the majority of whom were in high schools, received education at the K-12 level in Türkiye, and more than 1 million teachers worked (MoNE, 2023). In the Republic of Türkiye, which recently celebrated its 100th anniversary, the number of students increased by 53 times, the number of teachers by 93 times, and the number of schools by 15 times (MoNE, 2024a). These data indicate that the Turkish education system is a macro system (N. Özdemir et al., 2023a). Reform efforts aimed at improving this macro system have been adopted as one of the fundamental policies since the foundation of the republic (MoNE, 2017; MoNE, 2024a). For example, the “professional standards” determined for school administrators and teachers in Türkiye (MoNE, 2017) envisage them to be innovative individuals who adapt to the changes of the age. The “3 basic competencies” (professional knowledge, professional skills, attitudes and values) that teachers are expected to have emphasize the continuity and quality of teachers’ professional learning and their contribution to student success. The “school-based professional development model” aims to integrate the professional development of administrators and teachers on a school basis (MoNE, 2010). In addition, a reform discussion is currently underway, “The Century of Türkiye Education Model”, which aims to simplify the curriculum (MoNE, 2024b). However, the improvement efforts made on this large student and teacher population of the Turkish education system often result in expectations not being met.

#### 1.1.5. The Relationship Between Innovative School Leadership and Teacher Professional Learning

There is an increasing body of knowledge about the relationship between school leadership and TPL in terms of some leadership models (Thien & Yeap, 2023). Less is known about the relationship between TPL and current leadership models such as ISL. However, some evidence suggests that ISL and TPL are related (Khanthap, 2022; Valenzuela & Callo, 2024). In addition, Karacabey et al. (2020) conducted a study with a large dataset obtained from Turkish teachers and found that transformational leadership and TPL were significantly related. Relying on the fact that innovative school leadership originates from transformational leadership (Bass & Riggio, 2006) and other results in the literature, we predicted a positive relationship between ISL and TPL. Therefore, we proposed our hypothesis H1 in our research.

**H1.**  *There is a positive significant relationship between ISL and teacher professional learning.*

#### 1.1.6. Innovative School Leadership and Principal–Teacher Relationship

Innovative school leaders are those who can establish positive connections with teachers, create highly effective teams with their communication skills, and enable teachers’ great ideas to emerge (Cunniff et al., 2013; Davis, 2019; Gonzales et al., 2024). ISL positively affects teachers’ emotional characteristics such as motivation and satisfaction (Orphanos & Orr, 2014) and behavioral characteristics such as entrepreneurship (Akyürek & Göktaş, 2023) and performance (Valenzuela & Callo, 2024). In our research, we observed that teachers’ high motivation and satisfaction feelings and high performance and entrepreneurial behavior will be associated with the context of positive principal–teacher relationships. In addition, Karacabey et al. (2020) determined in their research that transformational leadership, which provides the theoretical background to ISL, has a higher effect size on teacher trust, which is also organically linked to PTR, than a different leadership model (educational). The fact that the model that included transformational leadership and leader–member exchange in the study of Vermeulen et al. (2022) were very compatible increased our belief that ISL and PTR would be positive. Therefore, we proposed our H2 hypothesis in our research.

**H2.**  *There is a positive significant relationship between innovative school leadership and the principal–teacher relationship.*

#### 1.1.7. Teacher Professional Learning and the Teacher-Principal Relationship

Schools are learning centers for teachers as well as students. Teachers conduct most of their daily professional learning in schools (Kwakman, 2003; Vermeulen et al., 2022). Therefore, school principals are the key figures for school development and student and teacher learning. Indeed, the legal texts of education policies in most countries emphasize the role of school principals in achieving national goals (Bush, 2024). Studies point to the link between transparent, supportive and fair relationships that school principals establish with teachers and TPL. For example, the influencers of TPL were determined as school principals’ sharing of professional knowledge (Thien & Yeap, 2023), fair operation of school procedures by principals (Liu & Hallinger, 2024), ethical behaviors (Liu & Yin, 2023), and the quality of communication between principals and teachers (Thien et al., 2024). In addition, Karacabey et al. (2020) determined a direct relationship between teacher confidence and TPL, which can provide important information about PTR. With this support from the literature, we propose that PTR and TPL are related in our H3 hypothesis.

**H3.**  *There is a positive and significant relationship between the principal–teacher relationship and teacher professional learning.*

#### 1.1.8. The Mediating Role of the Principal–Teacher Relationship on the Relationship Between Innovative School Leadership and Teacher Professional Learning

Previous studies in the field of educational administration and leadership have provided sufficient and convincing evidence about the positive relationship between school leadership and TPL (Liu et al., 2016; Thien et al., 2024). More specifically, several studies have shown that the innovative leadership characteristics of school principals are associated with TPL (Karacabey et al., 2020; Khanthap, 2022; Valenzuela & Callo, 2024). In addition, studies have found that there are mediating mechanisms that link leadership and TPL, such as teacher obligation (Liu & Yin, 2023), teacher agency (Liu et al., 2016), trust (Liu et al., 2016; Talebizadeh et al., 2021), and knowledge sharing (Talebizadeh et al., 2021). In addition, there are results in the literature showing that positive interaction between school principals and teachers, in other words, PTR, affects both school leadership and TPL (Karacabey et al., 2020; Thien et al., 2024). Taking into account ISL’s status quo-challenging, future-oriented (Alharbi, 2021), and TPL-supportive approach (Khanthap, 2022; Valenzuela & Callo, 2024), we propose with our hypothesis H4 that the principal–teacher relationship will mediate the relationship between ISL and teachers’ professional learning.

**H4.**  *The principal–teacher relationship mediates the relationship between ISL and teacher professional learning.*

## 2. Materials and Methods

### 2.1. Research Model

The study was conducted using quantitative methods to determine the relationships between ISL, PTR, and TPL using correlational, cross-sectional, and mediation design (Cohen et al., 2000). The mediation model in Figure 1 was tested within the scope of the study.

### 2.2. Universe and Sample

In order to conduct the research, ethics committee approval (Kütahya Dumlupınar University Social and Human Sciences Scientific Research and Publication Ethics Committee’s letter dated 15 April 2024, and numbered 127) was obtained.

The universe of the research consists of 2687 teachers working in high schools in the Odunpazarı and Tepebaşı districts of Eskişehir province of Türkiye (MoNE, 2023). According to the 95% confidence interval, the lower limit for the sample size of the study is 336. The sample consists of 357 teachers working in high schools in these districts in the 2023–2024 academic year. The sample size is sufficient according to the 95% confidence interval according to the universe in this study. The sampling of teachers was carried out using the simple random sampling method. Random selection was carried out in an objective manner among existing schools and teachers. Descriptive statistics regarding demographic variables are given in Table 1.

The table shows that female teachers are more represented than male teachers with 65.3% and in terms of the education status variable, there are more bachelor’s degree teachers than postgraduate graduate teachers with 76.5%. For the teaching seniority variable, the group with the highest rate is “16 years and above” with 64.1%, and the group with the lowest rate is “1–5 years” with 3.4%; according to the tenure variable in the school, the group with the highest rate is “1–3 years” with 35.3%, and the group with the lowest rate is “4–6 years” with 16%.

### 2.3. Data Collection Tools

In order to carry out the necessary applications and analyses on the research topics, scales with verified validity and reliability were used. These are the Innovative School Leadership Scale, Teacher Professional Learning Scale, and Principal–Teacher Relationship Scale. The first scale, which consists of 28 items to measure ISL level, consists of a single theoretical dimension. The second scale consists of 27 items and four dimensions (collaboration, reflection, implementation, and access to knowledge base) to measure TPL level. The last scale consists of 10 items and two dimensions (closeness and conflict), aiming to measure the PTR level.

#### 2.3.1. Innovative School Leadership Scale

“The Innovative School Leadership Scale” (ISLS) developed by Akyürek and Karabay (2022) was used to determine ISL in the study (Sample items: Provides enough time for teachers to generate innovative ideas/Encourages teachers to generate innovative ideas/Empowers teachers to be innovative). The ISLS is a five-point Likert-type scale (1 = Strongly disagree, 5 = Strongly agree). The scale, consisting of 28 items aiming to measure the ISL level, was developed based on a single theoretical dimension. As a result of the confirmatory factor analysis (CFA) conducted to verify the factor pattern of the tool, t values were found to be significant at the 0.01 level. Thus, all indicators were included in the model. The CFA results are shown in Table 2.

When the table is examined, the *p* value is significant at the 0.01 level. It is normal for the *p* value to be significant due to the large sample in most CFAs. For this reason, alternative fit indices were examined. As a result of the analyzes, X^2^/sd, SRMR, NNFI, and CFI values were excellent; RMSEA and GFI values were found to have good fit. In this context, the 28-item and one-dimensional structure of the scale was confirmed as a model. The reliability analysis of the scale was performed using item-total correlation and Cronbach alpha, and the results are presented in Table 3.

The overall internal consistency coefficient (Cronbach’s alpha) of ISLS is 0.99. This value is sufficient for the reliability of the scale scores. The item-total correlations for all items in the scale range from 0.70 to 0.93. This indicates that the scale items discriminate well among individuals.

#### 2.3.2. Teacher Professional Learning Scale

In the study, the Teacher Professional Learning Scale (TPLS), developed by Liu et al. (2016) and adapted to Turkish by Gümüş et al. (2018a), was used to determine TPL. The scale is a five-point Likert-type scale (1 = Strongly disagree, 5 = Strongly agree). The scale consists of 4 dimensions and 27 items in total: collaboration (6 items/sample items: I share my teaching experiences with my colleagues), reflection (10 items/sample items: I organize my teaching methods according to students’ responses), implementation (5 items/sample items: I apply new teaching methods in my lessons), and accessing the knowledge base (6 items/sample items: I collect feedback from students about their learning). As a result of the CFA conducted to verify the factor design of the scale, t values were found to be significant at the 0.01 level. Thus, all indicators were included in the model. The CFA results are shown in Table 4.

When the table is examined, the *p* value is significant at the 0.01 level. It is normal for the *p* value to be significant due to the large sample in most CFAs. Therefore, alternative fit indices were examined. As a result of the analyzes, it was found that the X^2^/sd and SRMR values had excellent fit; the RMSEA and CFI values had good fit; GFI and NNFI values were close to good fit. In this context, the 27-item and four-dimensional structure of the scale was confirmed as a model. The reliability analysis of the scale was performed using item-total correlation and Cronbach alpha, and the results are presented in Table 5.

The general internal consistency coefficient (Cronbach alpha) of TPLS is 0.95. This value is sufficient in terms of the reliability of the scale scores. The item-total correlations for all items in the scale vary between 0.53 and 0.73. In this case, the scale items discriminate individuals well.

#### 2.3.3. Principal–Teacher Relationship Scale

The Principal–Teacher Relationship Scale (PTRS), developed by Zee et al. (2023) and adapted to Turkish by Akyürek and Akkoyun (2023), was used to determine PTR in the study. The scale is a five-point Likert-type scale (1 = Strongly disagree, 5 = Strongly agree). The scale consists of 2 dimensions and 10 items: closeness (5 items/sample item: My principal values his/her communication and interaction with me) and conflict (5 items/sample item: I feel that my principal does not treat me fairly). As a result of the CFA conducted to verify the factor design of the tool, t values were found to be significant at the 0.01 level. Thus, all indicators were included in the model. The CFA results are shown in Table 6.

When the table is examined, the *p* value is significant at the 0.01 level. It is normal for the *p* value to be significant due to the large sample in most CFAs. Therefore, alternative fit indices were examined. As a result of the analyzes, it was determined that the X^2^/sd, RMSEA, SRMR, GFI, NNFI, and CFI values had perfect fit. In this context, the 10-item and two-dimensional structure of the scale was verified as a model. The item-total correlation was used to analyze the reliability of the research, and the scale was analyzed using Cronbach’s alpha, and the results are presented in Table 7.

The general internal consistency coefficient (Cronbach’s alpha) of the PTRS is 0.89. This value is sufficient in terms of the reliability of the scale scores. The item-total correlations for all items in the scale vary between 0.43 and 0.77. This indicates that the scale items discriminate well among individuals.

The measurement tool used in the study was applied between 15 April–24 May, 2024. In the analyses, firstly, the normality assumption of the data set was evaluated and the standard deviation, skewness-kurtosis coefficients, and mean, median, and mode values were examined. The calculated standard deviation, skewness, and kurtosis values were 0.76, −1.10, and 1.89 in the ISL scale, 0.44, 0.20, and −0.30 in the teacher professional learning scale, and 0.74, −0.58, and 0.46 in the principal–teacher relationship scale. The kurtosis and skewness values in the study were between ±2. According to the results, the data set showed a normal distribution (George & Mallery, 2010). The calculated mean, median, and mode values were 4.08, 4.00, and 4.00 in the ISLS, 4.16, 4.07, and 4.00 in the teacher professional learning scale, and in the principal–teacher relationship scale, they were 3.87, 3.90, and 3.00. The closeness of the mean, median, and mode values also shows that the data set is normally distributed (Hair et al., 2011). In determining the relationships between the scores obtained from the scales within the normal distribution of the data set, the Pearson product-moment correlation coefficient was used.

A regression analysis based on the Bootstrap method was used to determine the mediation in the research model. The bootstrap method was preferred because it provides more robust estimates, especially when the sample size is limited. While traditional parametric methods can increase estimation errors in small samples, the bootstrap method calculates more robust standard errors and confidence intervals by resampling the data set (Mooney & Duval, 1993). The goal of the bootstrap method is to resample from the existing data set to produce very large data sets (Sacchi, 1998). The Bootstrap method provides more reliable results than the traditional method of Baron and Kenny (1986) and the Sobel test (Hayes, 2018; Zhao et al., 2010). In this context, the analyses were performed with the PROCESS Macro developed by Hayes (2018). In the analyses, 5000 resample options were used with the Bootstrap technique. In the mediation effect analyses conducted with the bootstrap technique, in order for the hypothesis to be supported, the 95% confidence interval (CI) values in the analysis results should not include the value zero (0) (MacKinnon et al., 2004). The data were analyzed with IBM SPSS 26, PROCESS Macro 4, and AMOS 22 package programs. In the interpretation of the analysis results, the 0.05 level was taken as the basis for statistical significance.

Cross-sectional collection of research data only from teachers may cause common method bias (Schaarschmidt et al., 2015). Therefore, Harman’s one-factor test was applied in the study to control this situation (Harman, 1967). All scale items were subjected to factor analysis with varimax rotation. The results obtained from this test showed that all items used explained only 43.47% of the total variance and did not exceed the 50% threshold, indicating that common method bias was not a serious problem in the study. In addition, we tried to reduce the common method bias by structuring the scale form as dependent, mediator, and independent variables respectively (MacKenzie & Podsakoff, 2012).

## 3. Results

Descriptive statistics of the scales within the scope of the study and the relationships between variables were analyzed, and the results are presented in Table 8.

The table shows that teachers’ perceptions of ISL ($\bar{x}$ = 4.08), TPL ($\bar{x}$ = 4.17), and PTR ($\bar{x}$ = 3.88) are at a high level. In addition, there are moderate positive significant relationships between ISL and TPL (r = 0.62, *p* < 0.01) and ISL and PTR (r = 0.42, *p* < 0.01) and a low positive significant relationship between TPL and PTR (r = 0.19, *p* < 0.01).

A regression analysis based on the Bootstrap method was performed to test the mediating role of PTR in the effect of ISL on TPL. The regression analysis results for the mediation test conducted for this purpose are given in Table 9.

When the table is examined, according to the H1 hypothesis test results of the research (in the absence of PTR), ISL has a positive significant relationship on TPL (b = 0.418, 95% CI [0.3874, 0.4486], t = 8.6685, *p* < 0.01). In this case, the H1 hypothesis is supported. According to the H2 hypothesis test results of the research, ISL has a positive significant relationship on PTR (b = 0.598, 95% CI [0.5186, 0.6785], t = 14.7235, *p* < 0.01). In this case, the H2 hypothesis is supported. According to the H3 hypothesis test results of the study, PTR has a positive significant relationship on TPL (b = 0.105, 95% CI [0.1764, 0.0343], t = 2.9155, *p* < 0.01). In this case, H3 hypothesis was supported.

**Figure 2:**
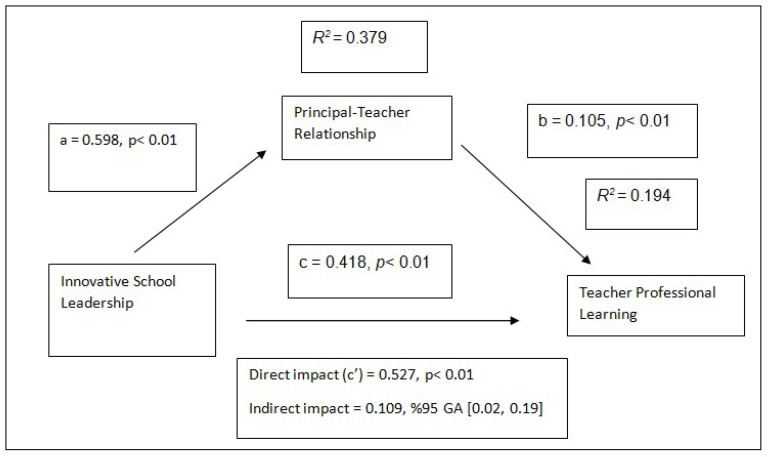
The mediating role of the principal–teacher relationship in the relationship between innovative school leadership and teacher professional learning (N = 357).

The indirect effect of ISL on TPL was determined according to the confidence intervals obtained with the Bootstrap technique. Accordingly, it was determined that the indirect effect of ISL on TPL was significant, and therefore PTR mediated the relationship between ISL and TPL (b = 0.109, 95% CI [0.0227, 0.1977]). The Bootstrap lower and upper confidence interval values obtained with the percentage method do not include the value 0 (zero). In this case, H4 hypothesis was supported. The regression analysis results regarding the mediation test are also shown on the model in Figure 2.

## 4. Discussion, Conclusions, and Recommendations

In this study, we theoretically demonstrated and empirically supported the hypothesis that ISL supports TPL through PTR. In this study, we theorized and empirically supported the hypothesis that ISL supports TPL through PTR, providing evidence to the knowledge base focusing on the relationship between school leadership and teacher professional learning (Liu & Yin, 2023; Liu et al., 2016; Thien & Yeap, 2023) through the lens of a different leadership model, ISL, and by demonstrating the mediating role of PTR, we provided evidence from Türkiye, an eastern society characterized by high power distance and low openness to uncertainty. Before discussing the findings of our study, we would like to explain some of the limitations of the study and recommend that our study be interpreted in line with these limitations.

### 4.1. Limitations

First of all, our research is a cross-sectional study conducted using quantitative methods. This means that while it provides a broad perspective on the relationships between the variables in the research, it will not provide an in-depth perspective on the dynamics of the variables. Longitudinal research should be conducted to determine the direction of the variables and qualitative research should be conducted to understand the depth of the variables. This is particularly important since we aimed to reveal the dynamics between school leadership and TPL in a different context (PTR). Another limitation is related to the sample of the research. Although we based our research on a strong theoretical background, we only tested our research model with 357 teachers working in high schools in the Eskişehir province of Türkiye. In addition, the design of our research and the fact that we obtained the data through online surveys did not allow us to make multilevel statistics. One of the limitations of the study is that the data used in CFA and hypothesis testing were obtained from the same sample. Since our data were obtained from scales based on participants’ self-reports, common method bias may have occurred in our study and the results may be affected by social desirability. To reduce this, we presented the survey forms in a ranking in which teachers evaluated school leaders and themselves. We took procedural precautions by not requesting identifying information about the participants in the survey forms. However, we may not have fully avoided common method bias and social desirability problems. Although Eskişehir, where the study was conducted, has characteristics that may reflect the general context of Türkiye and the distribution of teachers in the sample according to their gender is consistent with the general context of Türkiye (MoNE, 2023), the cultural context of the high schools and Eskişehir may limit the generalizability of the findings. Therefore, developing and testing the model with multilevel statistics including different variables that will mediate and moderate ISL and TPL with a sample of teachers working in different types of schools across Türkiye may provide more support for generalizability and expand knowledge in the field. In addition, we relied on teachers’ self-reports to assess PTR. We recognize this as an important limitation given the reciprocal nature of the relationships and recommend that principals’ perceptions be included in future research.

In addition, the fact that the study was conducted in an eastern society, in a centralized and bureaucratic educational system, requires caution in generalizing the results to other educational systems.

### 4.2. Discussion

Our findings first showed that the teachers who participated in the study perceived the ISL characteristics of school principals, their own professional learning, and their principals’ relationships with teachers at a high level. As in our study, Akyürek and Göktaş (2023) determined that teachers working in private schools in Türkiye perceived the ISL characteristics of their principals as high, and Al-Omari (2024) found that those working in public universities in Jordan had the same perceptions. Our findings are also consistent with other studies showing that teachers perceive the innovative leadership characteristics of school principals as high (Ariratana et al., 2019; Khanthap, 2022; Valenzuela & Callo, 2024).

Teachers’ high perceptions of school principals’ ISL characteristics are quite promising for the success of educational reforms because innovative school leaders have important positive effects such as positively affecting teachers’ entrepreneurial behaviors (Akyürek & Göktaş, 2023), their performance (Ghodang, 2021; Valenzuela & Callo, 2024), and the organizational happiness levels of schools (Akyürek & Göktaş, 2023). In fact, one of the important findings of school effectiveness research on this subject has been that some schools achieve more successful outcomes than others despite having similar conditions and resources (Cheng & Wong, 1996; Edmonds, 1979).

Conceptual studies emphasize some very important cognitive, affective, and behavioral characteristics and managerial practices that innovative leaders have, such as knowledge, skills, values, willpower, and cultural sensitivity (Alharbi, 2021; Davis, 2019). For example, Marron and Cunniff (2014) emphasized that the characteristics of an innovative school leader, such as heart, empathy, learning, passion, perseverance, strategy, and pleasure, which resemble the character strengths and virtues of positive psychology (Peterson & Seligman, 2004), are characteristics that all school principals must possess. It is widely accepted that innovation is specific to individualistic societies characterized by low power distance and openness to uncertainty (Herbig & Dunphy, 1998; Hofstede, 1980; Kaasa & Vadi, 2010). In addition, it is noteworthy that the characteristics of innovative leaders emphasize not only adaptability to change but also humanistic factors based on beliefs and values linked to the ancient culture of humanity and leadership.

Cultures affect all social characteristics (Herbig & Dunphy, 1998) and shape the leadership approaches and PTR of school principals (Rockstuhl et al., 2012; S. Özdemir et al., 2023b). Hofstede (1980) classified Türkiye among the countries with high power distance and low openness to uncertainty in terms of its social characteristics. It is stated in the literature that countries with these characteristics are disadvantaged in terms of innovation and change capacity (Herbig & Dunphy, 1998; Kaasa & Vadi, 2010). There may be several possible reasons why Turkish teachers perceive the innovative leadership characteristics and PTR of school principals as high in the research. Firstly, the fact that ISL is one of the less studied leadership models in the literature and teachers’ low awareness of ISL may have caused such an interpretation (Akyürek & Karabay, 2022; Othman & Rahman, 2013). The second reason may be that culture is not as effective as thought in determining the innovative leadership characteristics and PTR of school principals. This prediction can be supported by the findings of Rockstuhl et al. (2012), who examined leader-member interaction in the context of culture and examined studies from 23 different countries, showing that transformational leadership and leader–member interaction produced the same results in different cultures. The third reason, which we also strongly believe, is related to the change in cultural characteristics. Although the change is not very rapid, culture changes gradually but continuously to meet the needs of society (Herbig & Dunphy, 1998). In the half-century that has passed since Hofstede’s (1980) impressive study, the world has witnessed global transformations and incredible technological changes. The effects of globalization and the spread of internet technology to the most remote corners of countries have increased the interaction of cultures with each other as well as the rapid flow of information (Volti & Croissant, 2024). Like all other societies, Türkiye has been affected by these developments and innovations. In this context, it can be said that societies with low openness to uncertainty, such as Türkiye, are more sensitive to innovative practices compared to the past (European Commission, 2024). With this finding of our research, we would like to draw attention to this point; since a lot of time has passed since Hofstede’s classification, we recommend that multidisciplinary studies be conducted to shed light on the changes in the cultural characteristics of countries and their differences in education systems and principal, student, and teacher behaviors.

The centralized and hierarchical structure of the Turkish education system can create some bureaucratic obstacles between school principals and teachers. However, due to the nature of life in schools and the fact that school principals in Türkiye are selected from among teachers (MoNE, 2023), PTR practically relaxes this hierarchy. In addition, the fact that Turkish school principals see themselves as administrative managers and tend to have a more paternalistic leadership approach (S. Özdemir et al., 2023b) leads school principals to focus on ensuring the functioning of the school and to establish close relationships with teachers. Therefore, it was determined in the study that teachers have a fair interaction based on love and respect with their principals and that this interaction makes teachers feel peaceful (Zee et al., 2023).

The research results show that teachers feel their professional learning is high. Although the range of professional learning activities that teachers can participate in is quite wide, research shows that teachers participate in some of these activities more frequently and in others less frequently (Kwakman, 2003; OECD, 2019). According to the Teaching and Learning International Survey 2018 report, Turkish teachers participated in activities such as peer observation, self-observation, and guidance at a much lower rate than their most successful European and Asian peers in PISA, and participated in consultancy studies where experienced teachers support new teachers and in professional learning studies where student evaluations are analyzed at a much lower rate than their peers in OECD countries (OECD, 2019). Therefore, while the high perception of teachers’ professional learning activities in the study is evaluated positively, the frequency and diversity of their participation in professional learning activities provide an idea about the quality of these activities. Therefore, the quality of professional learning activities that teachers participate in should be increased. Teachers should be encouraged to participate in TPL activities that can directly affect student outcomes.

On the other hand, teachers’ perceptions of principals’ ISL, their own TPL characteristics, and PTL can be considered as a contradictory result in the context of Türkiye. To explain, according to the latest international assessment reports, Türkiye is among the rising innovative countries that have made significant progress in terms of innovation capacity, especially since 2013. However, these reports also show that Türkiye’s innovation capacity faces various challenges in areas such as human resources, research systems, and digitalization (Dutta et al., 2024; European Commission, 2024). For example, Türkiye is far behind the European Union (EU), where it is a candidate country, in terms of producing qualified scientific output and attracting students from different countries for doctoral education. Moreover, the proportion of new graduates in science, technology, engineering, and mathematics is significantly lower than the EU average. This shows that there are significant challenges in accessing a workforce with digital skills (European Commission, 2024). Similarly, despite significant innovative initiatives in education in recent years (e.g., MoNE, 2024a), international student comparisons point to Türkiye’s significant educational challenges (OECD, 2023). In the study, teachers’ high perceptions of themselves and school administrators towards ISL, TPL, and PTR contradict these international comparisons of Türkiye. Senge (1990) explains this situation as the difference between the theories that people adopt and the theories they apply. In other words, since the results obtained in our study are based on teachers’ perceptions, this may be due to a kind of pseudomorphism between perception and reality. As a matter of fact, Doğuş (2024) emphasized that in many studies, it has been concluded that pre-service teachers, teachers, school administrators, and students in Türkiye consider themselves quite competent in terms of 21st century skills, but Turkish students get low results in PISA exams measuring 21st century skills, and this is due to the difference between perception and reality.

Secondly, our findings showed evidence of a moderate positive relationship between ISL and TPL, and also ISL and PTR. Moreover, a direct significant relationship was determined between ISL and TPL in the absence of PTR. These findings allowed us to accept our hypotheses H1 and H2. This finding confirms studies that reached similar results (Akyürek & Göktaş, 2023; Khanthap, 2022; Valenzuela & Callo, 2024) and shows that school administrators’ ISL behaviors concretely and directly affect teachers’ professional learning and positive PTR. In addition, we found a positive relationship between TPL and PTR, albeit at a low level. This finding allowed our H3 hypothesis to be accepted, but in our research, we actually expected a stronger relationship between PTR and TPL based on previous literature (Karacabey et al., 2020; Hallinger & Kulophas, 2019; Liu & Hallinger, 2024; Thien et al., 2024; Vermeulen et al., 2022). One of the important roles of principals as school leaders is to facilitate TPL (Hallinger & Kulophas, 2019). Principals can foster supportive environments with caring relationships with teachers, create a safe and open school climate (Zee et al., 2023), and create a school environment that will support the development of a sense of responsibility among teachers and encourage them to engage in professional learning (Liu & Hallinger, 2024).

The sensitivity of educational practices to organizational structures and culture can lead to similar practices producing different results in different societies (Hallinger & Kulophas, 2019). For example, research from the Netherlands, where school administrators are held responsible for the quality of schools and known for their decentralized management structure and individualistic characteristics, has shown that leadership affects teachers’ professional learning, collaboration with colleagues, sense of well-being, and quality teaching practices (Thoonen et al., 2011). It has been pointed out that teachers’ participation in professional learning activities largely depends on their own personal characteristics and factors such as the type of school they work in (Kwakman, 2003; Vermeulen et al., 2022; Zee et al., 2023). The results from China, which has high centralization, power distance, and collectivist characteristics like Türkiye, emphasized the importance of trust among teachers (Liu et al., 2016), responsible behaviors (Liu & Hallinger, 2024), and ethical behaviors of school principals (Liu & Yin, 2023), in other words, collective characteristics rather than individuality.

We assume that the first possible reason for the low relationship we identified between PTR and TPL is that our study was conducted with high school teachers. It is likely that the confidence of high school teachers who are specialized in a field in their abilities makes their TPL needs less related to principal–teacher relationships because the study determined that high school teachers perceive both principal–teacher relationships and professional learning as high. In Türkiye, students prepare for a very challenging university exam at the end of their high school education. Especially in academic high schools, the main purpose of students continuing their education in these high schools is to be successful in the university exams and to study in one of the departments with high social prestige (e.g., medicine, law, engineering). Therefore, instead of positive PTR or TPL, teachers working in high schools prioritize being teachers who apply teacher-centered education methods and who can solve and teach students how to solve the best test questions (Kalaycıoğlu, 2015). Another possible reason may be related to the school size of high schools (MoNE, 2024a). This prediction is consistent with the conclusion of Zee et al. (2023) that high school teachers establish less-close relationships compared to primary and secondary school teachers and with the findings that school types differentiate the principal–teacher relationship and that teachers interact less with school principals in more specialized and bureaucratic school levels. However, more interaction between principals and teachers is important for the sustainability of positive PTR and TPL. This is because strong professional and personal relationships between principals and teachers provide a positive working environment and increase the collective capacity of the school (Etxeberria et al., 2017; Karacabey et al., 2020; Y. Li et al., 2023; Scheerens et al., 2013). The results of different studies show that in schools with quality PTR, teachers’ competencies improve (Karacabey et al., 2020), they trust their principals and colleagues (Hendawy Al-Mahdy et al., 2024; Karacabey et al., 2020; Thien et al., 2023), and their commitment to their schools increases (To et al., 2023). Teachers’ feelings of trust shaped by PTR also play a key role in their degree of engagement in TPL. Thus, teachers engage in more TPL activities when they feel trust (Karacabey et al., 2020). On the contrary, teachers may become alienated from their schools and professions (Stone-Johnson, 2016). Considering the vital role of TPL on school effectiveness and student learning (Karacabey et al., 2020; L. Li et al., 2016; Thien et al., 2023), a low relationship between PTR and TPL may make it difficult to improve teaching practices and increase student achievement, which is the ultimate goal of TPL (Chen, 2024). We expect that causal studies that will deeply investigate this low relationship between the principal–teacher relationship and TPL in future studies and develop understanding on this issue will provide more enlightening information.

Third, our findings showed that PTR mediated the relationship between ISL and TPL. This led to the acceptance of our hypothesis H4. To the best of our knowledge, our study is the first attempt to use PTR as a mediator of the ISL–TPL relationship. Therefore, we cannot provide direct empirical support for our hypothesis from the literature. However, we can support this hypothesis with previous studies in the field of educational administration and leadership. For example, Liu et al. (2016) revealed that relational trust is an important explanatory mechanism linking leadership and teacher learning. Talebizadeh et al. (2021) showed that when principals create an environment where positive relationships are established, such as developing trust in schools and encouraging knowledge sharing, teachers’ professional learning can be enhanced.

In our research, which we conducted by using teachers’ perceptions, our findings generally showed that PTR and TPL were related to ISL characteristics such as principals’ ability to guide and lead innovation, change and development in their schools and to create an innovative vision for their schools. These results provided important insights into the interactions and school practices of principals and teachers (Doran, 2004; Reid, 2020), the two most important factors affecting student outcomes. The most fundamental role of schools is to ensure student learning. Especially since the beginning of the new century, there has been an increasing demand from all sectors of society for schools to improve students’ learning as well as to develop them in multiple ways. Students are expected to be curious, enthusiastic, creative, and innovative individuals who can use what they learn at school to solve real-life problems. These demands, which constantly expand the roles of principals and teachers, increase the pressure on schools (Fullan, 2010; Pellegrina & Hilton, 2012; Reid, 2020). The innovative leadership qualities of school principals, the quality of their relationships with teachers, and the quality professional learning activities of teachers can make a difference in meeting these demands. By supporting teachers to use new approaches in their classrooms, providing resources to produce innovative products, and encouraging them to learn about innovations in teaching and learning, ISL has the potential to transform schools into learning centers for both students and teachers (Akyürek & Karabay, 2022). Good relationships and positive interactions between principals and teachers can lead to a school culture that supports change and innovation (Edwards-Groves et al., 2016; Galdames-Calderón, 2023). Thus, principals have the potential to improve student outcomes and increase the effectiveness of their schools by influencing teachers’ attitudes and behaviors (Bellibaş et al., 2022; Hallinger et al., 2020; Hallinger & Kulophas, 2019; L. Li et al., 2016). For example, innovative school leaders can facilitate teachers’ incorporation of technological developments such as artificial intelligence into classroom practices with their inherent innovativeness (Roberts & Richardson, 2024). Based on the relationships we found between ISL, TPL, and PTR in our study, we imply that ISL is important in improving student outcomes and enhancing school effectiveness.

### 4.3. Theoretical Implications

Our research showed the mediating role of PTR in the relationship between principals’ ISL and TPL. This revealed a mediating mechanism in the relationship between ISL and TPL. Although there are studies showing the relationship between ISL and TPL, it is important to empirically confirm that PTR is the mediator in this relationship. Thus, the research has filled this gap in the literature by identifying PTR as an important mediator between ISL and TPL and has become an important source of information for future research. Moreover, ISL can be expected to increase school effectiveness and thus improve student outcomes. It is possible that other variables other than PTR play a mediating and moderating role between ISL and TPL. We suggest that future research should examine different variables as mediators and moderators. For example, collective teacher innovativeness and teacher leadership may be two of them due to their capacity for innovation. Also, teacher trust is very important for TPL. Teacher trust can be examined as a mediating variable in the relationship between ISL and TPL.

### 4.4. Practical Implications

The findings of the study shed light on the intricate relationships between school leadership and TPL. This means that the positive relationships between principals and teachers are significantly related to the innovative methods that principals implement (e.g., using social networks effectively, encouraging teachers to use social networks to internationalize the school) and have the potential to improve TPL in schools. This indirect path emphasizes the importance of positive PTR in increasing principals’ innovativeness and improving teaching and student outcomes by increasing teachers’ professional competence. In our research, which we conducted based on teachers’ perceptions, our findings generally showed that PTR and TPL are related to ISL characteristics such as principals’ guiding and leading innovation, change, and development in their schools and creating an innovative vision for their schools. However, school principals may be limited in terms of legislation and bureaucratic reasons. For this reason, we think that school principals should be given more authority and autonomy in countries with centralized education systems. We also know from the literature that when school principals are trained with ISL characteristics, this has significant effects on their leadership characteristics and teachers’ collaboration with their colleagues (Orphanos & Orr, 2014). Therefore, we recommend developing programs for school principals in the Turkish education system, which does not implement a specific school leadership program, and including ISL skills in these programs. In this way, ISL’s capacity for innovation can be leveraged to facilitate the healthy adaptation of students, the citizens of the 21st century, to the technological changes that have characterized recent years, such as artificial intelligence. ISL can also make technological inclusion part of the school culture and teachers’ classroom practices and lessons can become more attractive to students. In addition, the Turkish Century Maarif Model, which is currently being implemented in Türkiye and is described as the biggest reform initiative of the Turkish education system, can be supported by school principals with ISL traits to increase its chances of success. We believe that our results will shed light on social structures and educational systems characterized by similar cultural characteristics (e.g., Azerbaijan), centralized structure (e.g., China, Oman, South Africa, Iran), large power distance, and strong uncertainty avoidance (e.g., Brazil, Colombia, Chile, Venezuela).

## Figures and Tables

**Table 1:** Descriptive statistics regarding demographic variables.

Variables		*N*	*%*
Gender	Female	233	65.3
	Male	124	34.7
Education status	Bachelor’s	273	76.5
	Postgraduate	84	23.5
Teaching seniority	1–5 years	12	3.4
	6–10 years	49	13.7
	11–15 years	67	18.8
	16 years and above	229	64.1
Tenure at the school	1–3 years	126	35.3
	4–6 years	57	16.0
	7–9 years	64	17.9
	10 years and above	110	30.8
Total		357	100

**Table 2:** Confirmatory factor analysis results of the innovative school leadership scale.

Measures of Compliance	Measurement Value	Reference Range
*p*	0.00	<0.01
X^2^/sd	2.38	≤3
RMSEA	0.06	≤0.06
SRMR	0.02	≤0.05
GFI	0.90	≥0.90
NNFI	0.97	≥0.95
CFI	0.98	≥0.95

**Table 3:** Reliability analysis results of the innovative school leadership scale.

Dimensions	Alpha Value	Item-Total Correlation
Scale	0.99	0.70–0.93

**Table 4:** Confirmatory factor analysis results of the teacher professional learning scale.

Measures of Compliance	Measurement Value	Reference Range
*p*	0.00	<0.01
X^2^/sd	3.00	≤3
RMSEA	0.07	≤0.07
SRMR	0.05	≤0.05
GFI	0.85	≤0.90
NNFI	0.88	≤0.90
CFI	0.90	≥0.90

**Table 5:** Reliability analysis results of the teacher professional learning scale.

Dimensions	Alpha Value	Item-Total Correlation
Collaboration	0.88	0.63–0.73
Reflection	0.86	0.46–0.69
Application	0.91	0.71–0.84
Access to knowledge base	0.81	0.52–0.66
Scale	0.95	0.53–0.73

**Table 6:** Confirmatory factor analysis results of the principal–teacher relationship scale.

Measures of Compliance	Measurement Value	Reference Range
*p*	0.00	<0.01
X^2^/sd	1.51	≤3
RMSEA	0.03	≤0.05
SRMR	0.03	≤0.05
GFI	0.98	≥0.95
NNFI	0.99	≥0.95
CFI	0.99	≥0.95

**Table 7:** Reliability analysis results of the principal–teacher relationship scale.

Dimensions	Alpha Value	Item-Total Correlation
Closeness	0.96	0.86–0.89
Conflict	0.87	0.52–0.83
Scale	0.89	0.43–0.77

**Table 8:** Descriptive statistics of the scales and correlation coefficients between variables.

Variables	Descriptive Statistics		Correlation Coefficients		
	$\bar{x}$	*SD*	1	2	3
Innovative School Leadership	4.08	0.76	---	0.42 *	0.62 *
Teacher Professional Learning	4.17	0.44		---	0.19 *
Principal–Teacher Relationship	3.88	0.74			---

* *p* < 0.01, $\bar{x}$: arithmetic mean, SD: standard deviation.

**Table 9:** Regression analysis results for the mediation test.

Prediction Variables	Outcome Variables					
	Principal–Teacher Relationship			Teacher Professional Learning		
	*b*	*p*	*S.E.*	*b*	*p*	*S.E.*
Innovative School Leadership	0.598 ***	0.000	0.025	0.305 ***	0.000	0.035
Principal–Teacher Relationship	---	---	---	0.105 **	0.003	0.036
Fixed	1.435 ***	0.000	0.168	3.327 ***	0.000	0.126
	*R*^2^ = 0.379			*R*^2^ = 0.194		
	*F*(1; 355) = 216.782; *p* < 0.001			*F*(2; 354) = 42.6156; *p* < 0.001		

Note: ** *p* < 0.01, *** *p* < 0.001; S.E.: standard error. Unstandardized beta coefficients (b) are reported.

## Data Availability

Data are available from the author upon reasonable request.
